# Human ACE2-Functionalized Gold “Virus-Trap” Nanostructures for Accurate Capture of SARS-CoV-2 and Single-Virus SERS Detection

**DOI:** 10.1007/s40820-021-00620-8

**Published:** 2021-04-13

**Authors:** Yong Yang, Yusi Peng, Chenglong Lin, Li Long, Jingying Hu, Jun He, Hui Zeng, Zhengren Huang, Zhi-Yuan Li, Masaki Tanemura, Jianlin Shi, John R. Lombardi, Xiaoying Luo

**Affiliations:** 1 State Key Laboratory of High-Performance Ceramics and Superfine Microstructures, Shanghai Institute of Ceramics, Chinese Academy of Sciences, 1295 Dingxi Road, Shanghai, 200050 People’s Republic of China; 2 Graduate School of the Chinese Academy of Sciences, Beijing, 100049 People’s Republic of China; 3 Center of Materials Science and Optoelectronics Engineering, University of Chinese Academy of Sciences, Beijing, 100049 People’s Republic of China; 4 School of Physics and Optoelectronics, South China University of Technology, Guangzhou, 510640 People’s Republic of China; 5 State Key Laboratory of Oncogenes and Related Genes, Shanghai Cancer Institute, Renji Hospital, Shanghai Jiaotong University School of Medicine, Shanghai, 200032 People’s Republic of China; 6 Anhui Provincial Center for Disease Control and Prevention, Hefei, 12560 Anhui People’s Republic of China; 7 Public Health Research Institute of Anhui Province, Hefei, 12560 Anhui People’s Republic of China; 8 Shanghai Yangpu Hospital of Traditional Chinese Medicine, Shanghai, 200090 People’s Republic of China; 9 Department of Frontier Materials, Nagoya Institute of Technology, Nagoya, 466-8555 Japan; 10 Department of Chemistry, The City College of New York, 160 Convent Avenue, New York, NY 10031 USA

**Keywords:** SERS, SARS-CoV-2, Human ACE2, “Virus-trap” nanostructure, Single-virus detection

## Abstract

The current COVID-19 pandemic urges the extremely sensitive and prompt detection of SARS-CoV-2 virus. Here, we present a Human Angiotensin-converting-enzyme 2 (ACE2)-functionalized gold “virus traps” nanostructure as an extremely sensitive SERS biosensor, to selectively capture and rapidly detect S-protein expressed coronavirus, such as the current SARS-CoV-2 in the contaminated water, down to the single-virus level. Such a SERS sensor features extraordinary 10^6^-fold virus enrichment originating from high-affinity of ACE2 with S protein as well as “virus-traps” composed of oblique gold nanoneedles, and 10^9^-fold enhancement of Raman signals originating from multi-component SERS effects. Furthermore, the identification standard of virus signals is established by machine-learning and identification techniques, resulting in an especially low detection limit of 80 copies mL^−1^ for the simulated contaminated water by SARS-CoV-2 virus with complex circumstance as short as 5 min, which is of great significance for achieving real-time monitoring and early warning of coronavirus. Moreover, here-developed method can be used to establish the identification standard for future unknown coronavirus, and immediately enable extremely sensitive and rapid detection of novel virus.

**Supplementary Information:**

The online version contains supplementary material available at 10.1007/s40820-021-00620-8.

## Highlights

- Extremely sensitive and prompt COVID-19 SERS biosensor to detect SARS-CoV-2 virus in the contaminated water at single-virus level.
- SERS sensor features a low detection limit of 80 copies mL^−1^ for the simulated contaminated water by SARS-CoV-2 virus as short as 5 min.
- ACE2-modified SERS sensor with machine-learning and identification standard enable rapid detection of novel yet-unknown coronaviruses.

**Supplementary Information:**

The online version contains supplementary material available at 10.1007/s40820-021-00620-8.

## Graphical abstract

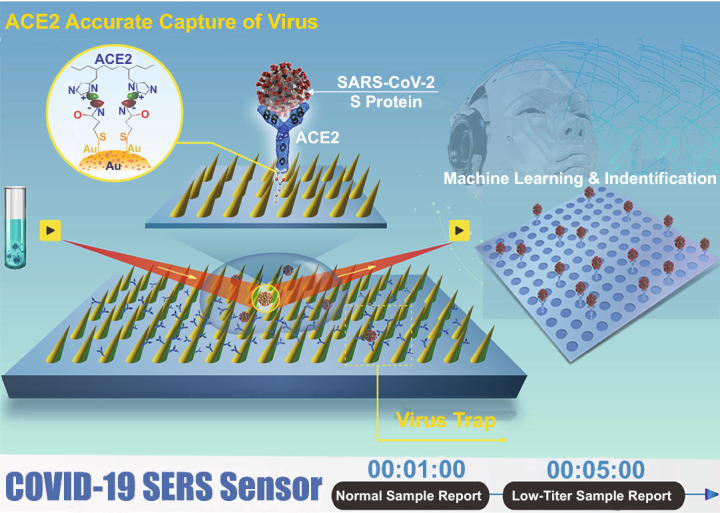

## Introduction

Severe acute respiratory syndrome-coronavirus 2 (SARS-CoV-2) is a positive strand RNA virus that causes severe respiratory syndrome in humans [1, 2]. The resulting outbreak of coronavirus disease 2019 (COVID-19) has emerged as a severe pandemic. More than 62 million confirmed cases including 1.46 million deaths around 200 countries have been reported to the World Health Organization (WHO) since December 2019 [3]. The major transmission routes are believed to be exposure to SARS-CoV-2-containing droplets or contaminated fomites. Therefore, there is an urgent public health request for rapid and accurate identification of newly emerging SARS-CoV-2 strains.

At present, SARS-CoV-2 has been identified from COVID-19 patients’ sputum, saliva, stool, serum, and urine with the viral load of 10^2^–10^7^ copies mL^−1^ [4–7]. It is worth noting that the SARS-CoV-2 virus shed from the fluids of symptomatic or asymptomatic patients may enter wastewater systems, which can remain infectious for up to 17–31 days [8, 9]. Therefore, in order to prevent the spread of the epidemic, it is much significant to detect and early warn the SARS-CoV-2 virus in the water environment of crowd gathering places. At present, Enzyme-linked immunosorbent assay (ELISA) [10] and real-time polymerase chain reaction (RT-PCR) [3, 7] analysis have been used to diagnose SARS-CoV-2 infection, and the typical sensitivity that can be achieved by RT-PCR is 500 ~ 1000 copies mL^−1^ of viral RNA [4, 11, 12]. However, these ELISA and PCR process suffer from complicated labour-intensive steps, as well as time (≥ 2 h) and high cost. Another powerful and promising tool involves clustered regularly interspaced short palindromic repeats (CRISPR) technology, which is being quickly deployed in the molecular diagnostics landscape. Mammoth Biosciences has developed a rapid (< 40 min), easy-to-implement and accurate CRISPR-based assay for the detection of SARS-CoV-2 from respiratory swab RNA extracts called the SARS-CoV-2 DNA Endonuclease-Targeted CRISPR Trans Reporter (DETECTR) [13]. Furthermore, many antibody test reagents are rapidly being developed and made available on the market to assist in the diagnosis of SARS-CoV-2 infection, including kits for testing total *Abs, IgM* antibody or *IgG* antibody, by chemiluminescence, ELISA or colloidal gold methods [14]. However, in the complex remote outdoor environment, the convenient, rapidly, economical, and high-sensitive virus detection is still a critical technical hurdle for outbreak preparedness.

Surfaced-enhanced Raman scattering (SERS) as a non-destructive, rapid and highly sensitive detection technique has exhibited the ability to detect single biomolecule such as DNA and proteins [15–24], been utilized to detect the respiratory viruses [25–28] including influenza viruses, adenovirus type 5 [29], and animal viruses [30] by engineered Au, Ag “hot-spots” plasmonic nanostructures. However, there are too many other proteins and biomacromolecules in the contaminated water by SARS-CoV-2 virus, such that these optical-engineered SERS-substrates suffer from overwhelming Raman signals from other impurities, leading to poor signal-to-interference ratio. Therefore, it is necessary to accurately capture the targeted virus first from SARS-CoV-2-containing droplets or contaminated fomites containing many impurities before SERS detection.

**Fig. 1:**
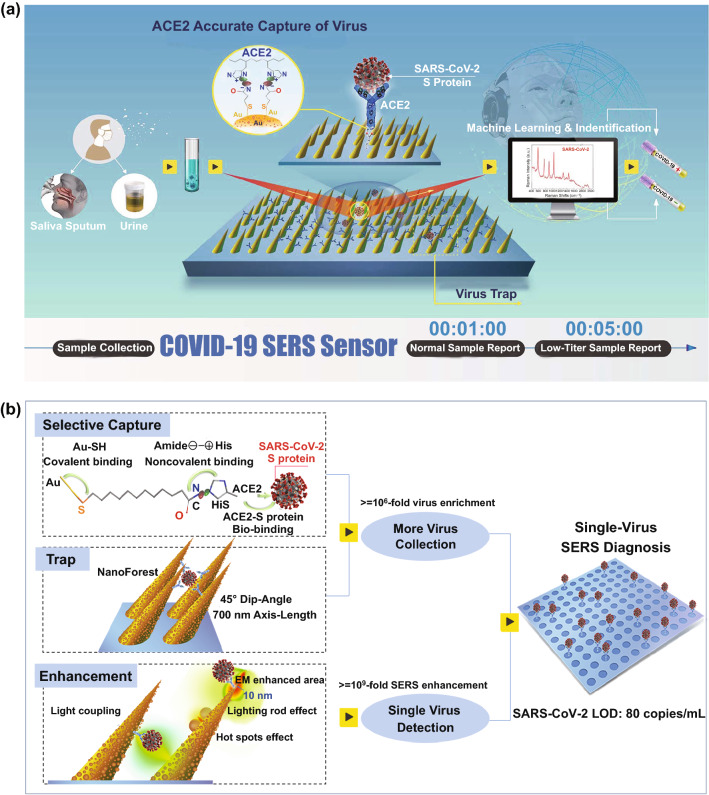
Schematic diagram of COVID-19 SERS sensor design and single-virus detection mechanism. **a** Schematic diagram of COVID-19 SERS sensor design and operation procedure. SARS-CoV-2 can be localized by “virus-traps” nanoforest composed of oblique gold-nanoneedles array (GNAs), and be captured by ACE2 anchored on amide-modified GNAs from virus-containing urines even with complex multi-proteins circumstance. Through machine-learning and identification techniques, the identification standard of virus signals are established, and utilized for virus diagnoses. **b** Schematic diagrams of single-virus detection by selectively capturing and trapping virus, and the multi-SERS enhancement mechanism

The spike glycoprotein (S protein) of coronavirus is a key target for vaccines, therapeutic antibodies, and diagnostics [31]. SARS-CoV-2 S and SARS-CoV S have been reported to share the same functional host cell receptor angiotensin-converting-enzyme 2 (ACE2). The higher affinity [31, 32] of SARS-CoV-2 S (10 ~ 20-fold than SARS-CoV S) for Human ACE2 may contribute to the significant ease of person-to-person transmission. Therefore, based on the higher binding ability of ACE2 with SARS-CoV-2 S, we propose a rapid and label-free ACE2-functionalized hierarchical gold nanoneedles array (GNA) as SERS sensor for selective capture and culture-free identification of SARS-CoV-2 in the contaminated water (Fig. 1a). The ACE2 is immobilized onto the amide-modified GNAs through the noncovalent interactions between amide (negative charge) and *His*-tagged (positive charge) ACE2 [33, 34]. ACE2 can effectively capture coronavirus from virus-containing droplets, and these oblique hierarchical GNAs can act like “virus-traps” to localize coronavirus into GNAs. The high affinity of ACE2 with S protein and the as-designed “virus-traps” nanoforest can synergistically help SERS sensors accurately capture most of SARS-CoV-2 from the contaminated water (Fig. 1b). Moreover, multicomponent SERS enhancements including “lighting-rod” effect on tips, “hot-spots” effect of adjacent gold nanoparticles, and light coupling effects in GNAs are designed to realize the high-sensitive detection of virus. Significantly, combined with machine-learning and identification techniques, single-virus diagnose can be realized in less than 5 min, and low detection limit (LOD) can be down to 80 copies mL^−1^.

## Materials and Methods

### Preparation of Gold Nanoneedles Array (GNA) and ACE2 Modification

GNAs were fabricated by an ion-beam sputtering technique to sputter gold film. For fabrication, the silicon wafers (001 crystallographic orientation and 1–10 Ω resistivity) (5 $\times$ 5 mm^2^, 0.5 $\times$ 0.5 mm^2^) were sonicated in acetone for 10 min. Then, the wafers were washed with deionized water and dried under nitrogen gas flow. Afterwards, the samples were transferred to piranha solution for 1 h. At last, the samples were washed with deionized water and dried with nitrogen gas. Au films with different thicknesses were deposited on pre-cleaned Silicon substrates by vacuum evaporation and the thickness of the Au film was controlled by evaporating time. The Au nanostructures were fabricated by an ion-beam system with a Kaufman-type Ar^**+**^-ion gun (Iontech. Inc. Ltd, model MPS 3000 FC). A thin carbon film was deposited on Au films prior to sputtering in order to enhance the ion-induced formation of nanostructures [22]. The incidence angle from the Ar^**+**^ ion beam was set at 45° to the surface of the Au film. The ion irradiation with the ion beam of 6 cm diameter and 1 keV energy was carried out at room temperature for 4–6 min. The basal and working pressures were 1.5 × 10^−5^ Pa and 2 × 10^−2^ Pa, respectively. The residue of the carbon layer was then removed by ethanol before characterization. The microstructure and morphology of GNAs were analysed by FEI Magellan 400 field emission scanning electron microscopy (FESEM).

The amide-modified GNAs [31] was fabricated by alkanethiolate-covered gold nanoneedles array, and then by the ligand exchange of hexanetiolate-coated gold nanoneedles with n-BuNHCO(C_10_H_20_)SH in CH_2_C_l2_ for 24 h with a 1:1 mol ratio of n-BuNHCO(C_10_H_20_)SH to C_6_H_13_S–Au; 20 μg ACE2-His (2.62 mg mL^−1^) in tube was dissolved in 1.50 mL PBS solution, then was diluted to the concentration of 0.2 μM. The amide-modified GNAs were immersed in ACE2-His PBS solution for at least 30 min for saturation adsorption.

### Protein Expression and the Protein Expressed Virus Strains Construct

Human ACE2 (2.62 mg mL^−1^), SARS-CoV spike glycoprotein (0.58 mg mL^−1^), SARS-CoV-2 spike glycoprotein (2.38 mg mL^−1^), SARS-CoV-2 nucleocapsid phosphoprotein (1.3 mg mL^−1^) were obtained from Sanyou Biopharmaceuticals Co. Ltd, obtained from 293 T protein expression system. They were stored in the refrigerator at − 80 °C.

Two kinds of SARS-CoV-2 spike protein and nucleocapsid protein packaging into lentivirus were constructed as a model. Two kinds of viral strains that encode the spike protein and nucleocapsid protein of SARS-CoV-2, called the V_S_ (SARS-CoV-2 spike glycoprotein were expressed, 3.3 × 10^7^ copies mL^−1^) and V_N_ (SARS-CoV-2 nucleocapsid phosphoprotein were expressed, CBV30002, 5.03 × 10^8^ copies mL^−1^) strain here, were obtained from COBIOER BIOSCIENCES CO., LTD. and PackGene Biotech, respectively. The inactivated SARS-CoV-2 virus (inactivated viral infectivity by heating at 56 °C for 30 min) were provided by Anhui Provincial Center for Disease Control and Prevention, and performed for SERS experiments in BSL-2 laboratory by portable Raman spectrometer (SEED 3000, Oceanhood). All proteins and virus were diluted by PBS to the designed concentration.

Participants who provided samples also provided written, informed consent to participate in this study. The Ethics Committee of the Shanghai Cancer Institute approved the study. All of the research was performed in China.

### SERS Experiments

As-prepared ACE2-functionalized GNAs were immersed in different SARS-CoV-2 S, SARS-CoV S, V_S_, and V_N_ in PBS solution or urine solution for different times, and taken out for washing by PBS-solution and drying. All the SERS spectra were collected by a Confocal Raman spectrometer (inVia, Renishaw, UK) under the excitation laser of 785 nm. For all measurements, × 50 objective, the spot size of 2 μm, the acquisition time of 1–2 s and the laser power of 60 mW were employed.

### Machine-Learning Method Based on PCA and DA

A total of 1094 Raman shifts from 550 to 1750 cm^−1^ were chosen as the variables for PCA (Principal Component Analysis) by using Python and Statistical Product and Service Solutions. The built-in “PCA” function was used to get principal component coefficients, principal component scores, and principal component variances. The first three principal components were acquired with F1 interpreting 84.66% of variances, F2 interpreting 6.41% of variances and F3 interpreting 1.06% of variances. The top two principal components contributed to 91.07% of cumulative contribution which was enough to distinguish the data. This meant 91.07% of information from original spectra was represented in the two-dimensional space. Loadings were performed with the eigenvector of covariance matrix for multiple sets of the sample Raman spectra data in comparison. Raman spectra were projected onto score plot proportionally to loadings. Error ellipses of 95% confidence were plotted by using the “error-ellipse” function. According to PCA, we can get the main difference of virus Raman spectra, then we classify these data obtained by PCA, and finally use DA (Discrimination Analysis) to classify the unknown samples based on classified data. The results show that we are able to discriminate the virus with PCA and DA.

## Results and Discussion

### Fabrication and Characterization of SERS Chips

**Fig. 2:**
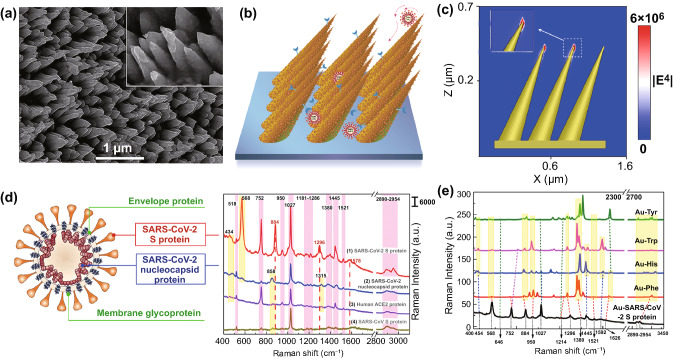
Morphology analysis of gold-nanoneedles array and SERS spectra of viral protein. **a** SEM images of Au nanoneedles array fabricated by Ar^**+**^ ions irradiation at a tilted angle of 45° on an Au film of 500 nm in thickness. **b** Schematics of “virus-traps” nanoforest composed of tilted gold-nanoneedles array. **c** Calculated intensity distribution (|E|^2^) at 785 nm for a tilted Au nanoneedle array with the polarization of the incident laser along the x-axis. **d** Structure schematics of SARS-CoV-2 (left), and SERS spectra (right) of SARS-CoV-2 S protein and nucleocapsid protein, SARS-CoV S protein, and Human ACE2 protein at 100 nM level. **e** Calculated static Raman spectra of 4 types of main individual amino acids *Tyr, Trp, His, Phe* encoded in SARS-CoV-2 S on Au cluster

Our SERS substrates consisting of 2D Au nanoneedles array with ordered arrangement were cost-effectively fabricated by oblique angle ion-beam sputtering techniques [22]. Figure 2a shows the SEM image of GNAs. The diameter of the nanoneedles in the stem part is around 300 nm, and the density of nanoneedles is approximately 9 μm^−2^. The axis lengths are around 700 nm, and the lengths of nanoneedles can be tuned from 300 to 1000 nm by sputtering gold films with different thickness. These nanoneedles are arranged into a two-dimensional array and arranged with an oblique angle of 45° to Si substrates. The tips of the nanoneedles exhibit an extremely sharp curvature with an apex diameter of 15 nm. The well-designed high-density nanoneedles with appropriate oblique angle and structural parameters can form “virus traps” nanoforest (Fig. 2b) to localize virus with the size of 50–100 nm, which can induce viruses to easily fall into nanoneedles array, obstruct these viruses from escaping by attachment to the tips. More interesting, these nanoneedles exhibit hierarchical nanostructures composed of many small gold nanoparticles with a diameter around 40 nm. These sharp tips can induce a “lighting-rod” effect (Fig. 2c) and accumulated nanoparticles can exhibit the “hot-spots” effect (Fig. S2), which can synergistically enhance the SERS effect. At the same time, the induced light in the array can engage in reflecting and scattering for many times, which can result in electromagnetic field (EM) coupling and further enhance the SERS effect [20, 21] with the enhancement factor of 10^9^ for R6G (Details, see S1), as shown in the simulated EM intensity distribution (Fig. S2) and movie (Movie S1) of GNAs.

### SERS Spectra of SARS-CoV-2 S Protein and Nucleocapsid Protein, SARS-CoV-S Protein, and Human ACE2 Protein

SARS-CoV-2 and SARS-CoV are belong to betacoronavirus, have single-positive strand RNA genome. Coronavirus genomes encode four structural proteins: spike (S), envelope (E), matrix (M), and nucleocapsid (N) [35]. In particular, the spike proteins overlap on the surface of coronavirus and exhibit amino acid sequence diversity among coronavirus, enabling the coronavirus anchored to human cell by the affinity binding to Human ACE2 protein with different binding strength. Surface protein and lipid profiles are distinctive characteristics of each virus. Usually, viruses can generate detectable characteristic Raman signals containing the surface protein and lipid profiles when their individual surface molecules are adequately in contact with metal nanoparticles, because viruses possess unique surface protein and lipid profiles on their outer layer [36, 37]. SARS-CoV-2 viruses are approximately 100 nm in diameter and thereby larger than the molecules that are conventionally analysable by SERS. In the case of SARS-CoV-2, the spike glycoprotein (S protein) on the virion surface mediates receptor recognition and membrane fusion [31, 38]. SARS-CoV-2 virus are covered by S proteins with about tens of nanometers in size. When their individual surface of SARS-CoV-2 virus are adequately in contact with metal nanoparticles, the surface S proteins will range in the “hot-spots” area and generate detectable characteristic Raman signals. Therefore, the detectable characteristic Raman signals usually contain the surface S protein will tend to dominate the SERS-Raman spectra of coronavirus.

Here, we initially characterized SERS spectra of four kinds of proteins: SARS-CoV-2 S protein, SARS-CoV-2 nucleocapsid protein, SARS-CoV S protein, and Human ACE2 protein, as shown in Fig. 2d (Details, see S2). These four kinds of proteins exhibited distinguishable Raman bands. In the region of 2920–2960 cm^−1^, overexpression of C-H vibrations and CH_2_ asymmetric stretch can be observed in four kinds of proteins [36, 38]. These three bands around 752, 1027, and 1445 cm^−1^ are possessed by all proteins, and can be attributed to Tryptophan (*Trp*), Phenylalanine (*Phe*) and CH_2_ deformation of Amino acid, respectively. While the characterized Raman bands for SARS-CoV-2 S protein including 568, 884, and 1296 cm^−1^, can be attributed to Amide V, *Trp* and Amide III (*α*-Helix) [36, 38, 39]. Interestingly, except for three unique characterized bands of SARS-CoV-2 S protein around 568, 884, 1296, and 1578 cm^−1^, almost all Raman vibrations in the Human ACE2 protein are contained in that of SARS-CoV-2 S protein. Figure 2e shows the calculated static Raman spectra of several amino acids on Au substrates. These bands around 568, 884, 950, 1027, and 1445 cm^−1^, can be found in the simulated Raman spectra of *Phe*, Histidine (*His*), *Trp*, Tyrosine (*Tyr*) [36, 38, 39], matching well with the experimental SERS spectra of SARS-CoV-2 S protein. SERS spectra of different proteins were then analysed using biomolecular Raman assignments from the literature [36, 38] in order to identify Raman peak assignments of the proteins (Table S1). The PCA (Principal Component Analysis) result (Fig. S3) shows that the individual S proteins in SARS-CoV-2 and SARS-CoV can be distinguished clearly, indicating SARS-CoV-2 and SARS-CoV can be identified by their SERS spectra. Additionally, we collected the SERS signals of SARS-CoV-2 S protein in the microscope regions with the area of 20 × 14 μm^2^, and the relative standard deviations (RSD) of two Raman vibration intensity (569 cm^−1^, 1027 cm^−1^) are 10.7% and 4.9% (Fig. S4), indicating the excellent SERS uniformity.

### Affinity of SARS-CoV-2 S, SARS-CoV S with ACE2

**Fig. 3:**
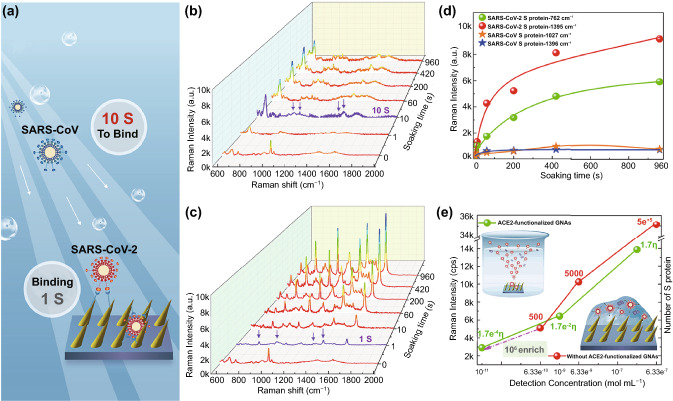
Affinity analysis of SARS-CoV-2 S, SARS-CoV S with ACE2. **a** Schematic illustration of S proteins’ binding on ACE2-functionalized GNAs of SARS-CoV-2 and SARS-CoV. SERS spectra of **b** SARS-CoV and **c** SARS-CoV-2 S proteins at the same concentration of 177 nM after bound to ACE2-functionalized GNAS for different binding time durations. **d** Intensity of detected SERS signals of SARS-CoV-2 S and SARS-CoV S after bound to ACE2-functionalized GNAs with different binding times. **e** Intensity of Raman bands (1027 cm^−1^) of SARS-CoV-2 S with different concentration detected with ACE2-functionalized GNAs by immersing in the diluted protein solution, and without ACE2-functionalized GNAs by dropping the corresponding concentration of diluted protein solution. It is worth noting that the application of dropping method is because GNAs without ACE2 modification have little affinity with SARS-CoV-2 S protein. The value marked on the line represents the number of S proteins in one Raman-focused window. η represents enrichment multiple by ACE2

The binding time between S protein and ACE2 was measured in order to find the appropriate time for ACE2-functionalized SERS chips to fully capture SARS-CoV-2-like virus. The SERS spectra of SARS-CoV-2 S and SARS-CoV S proteins with the same concentration of 177 nM after adsorbing on ACE2-functionalized SERS chips with different binding times, are shown in Fig. 3. Very surprisingly, when the ACE2-functionalized SERS chip was immersed in SARS-CoV-2 S PBS solution with such low concentration for just one second, the distinct Raman bands (highlight by the purple arrows in Fig. 3c) of SARS-CoV-2 S (762, 950, 1302, and 1395 cm^−1^) could be observed, suggesting that the process for SARS-CoV-2 viral infection was quite rapid [35, 40]. After 7 min, virus adsorption approached to saturation (Fig. 3d), indicating short pre-treatment time for detecting SARS-CoV-2-containing droplets. The Raman bands of SARS-CoV S were not obvious when ACE2-functionalized SERS chip was immersed in SARS-CoV S PBS solution just one second, became obvious (highlight by the purple arrows in Fig. 3b) only after binding for 10 s. After 7 min, virus adsorption also approached to saturation, but the intensities of characterized Raman bands for SARS-CoV S (762, 1027, and 1396 cm^−1^) were several-fold less than those of SARS-CoV-2 S (Fig. 3d). Above results demonstrated that the affinity of SARS-CoV-2 S binding to ACE2 was higher than SARS-CoV S, and the viral infection process for SARS-CoV-2 was more rapid than SARS-CoV. Furthermore, we checked the LOD of SARS-CoV-2 S by our ACE2-functionalized SERS chips (Figs. S5 and 3e), and found it was very sensitive with LOD down to 17.7 pM. While the LOD was 0.63 nM when SARS-CoV-2 S were dropped on GNAs without ACE2-modification. Based on above results, at least 10^6^-fold enrichment (enrichment multiple η, details, see S3) of SARS-CoV-2 S proteins can be obtained [33], far higher than the 75-fold enrichment using the physical enrichment method [27]. It can be attributed to most of S proteins tend to move to ACE2-functionalized GNAs, rather than randomly Brownian diffuse (Fig. 3e). It demonstrated that ACE2 selectively binding capability with S protein expressed on viral surface and oblique gold nanoneedles formed physical “virus-traps” nanostructures could play a vital role in accurately capturing and enriching SARS-CoV-2.

### SERS Spectra of Viral Strains Expressing SARS-CoV-2 Spike and Nucleocapsid Proteins, and Establishing Identification Standard

**Fig. 4:**
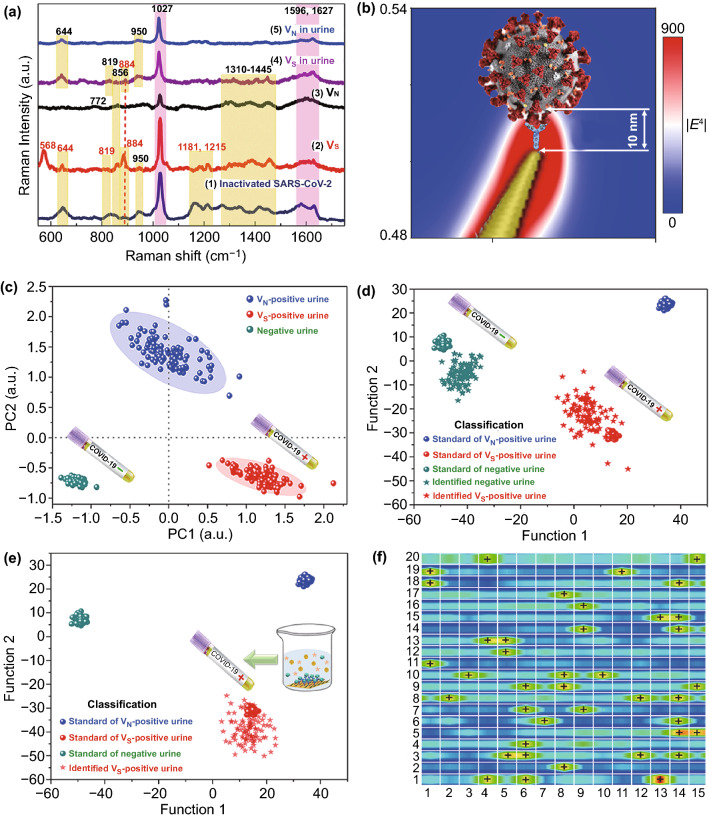
Establishing and validating the SARS-CoV-2 virus identification standard based on machine-learning method. **a** SERS spectra of inactivated SARS-CoV-2, V_N_ and V_S_ in PBS solution and urine of healthy 8-year-old girl patient with the viral load of 2200 copies/mL. Navy blue, red, black, purple and blue lines represent inactivated SARS-CoV-2, V_S_, V_N_, V_S_ in urine and V_N_ in urine, respectively. **b** Schematic of SARS-CoV-2 S localized within the 10 nm EM enhancement area, and calculated intensity distribution (|E|^2^) at 785 nm for an oblique Au nanoneedle’s tip. **c** Key features of SERS patterns to classify the urine samples infected by V_S_ (simulated contaminated water by SARS-CoV-2 virus), V_N_ and healthy people via PCA. **d** DA results to identify the urines for chronic nephritis and V_S_-containing chronic nephritis. The green, red, blue balls represent the standard of negative urine, the standard of V_S_-positive urine, the standard of V_N_-positive urine. The green and red stars represent identified negative urine and identified V_S_-positive urine. **e** DA results to identify V_S_ and V_N_ virus mixed in the adult’s urine (2200 copies mL^−1^). The green, red, blue balls represent the standard of V_S_-negative urine, the standard of V_S_-positive urine, the standard of V_N_-positive urine. The red star represents identified V_S_-positive urine. **f** SERS mapping (40 × 30 μm^2^) of 300 measuring area for one urine sample, 42 dot-measuring-area can be identified as V_S_-positive

SARS-CoV-2 exhibits very high infection ability and can lead to severe illness for researchers in the experiments, we must construct SARS-CoV-2-like virus that lack replication ability but still express the surface bioinformation of SARS-CoV-2. Therefore, two kinds of viral strains that encode the spike protein and nucleocapsid proteins of SARS-CoV-2 (Fig. S6), called the V_S_ and V_N_ strain here, were constructed in our experiments [30, 41]. The virus particles were approximately 50–60 nm in diameter and possessed roughly spherical shapes, as shown in Fig. S7. We obtained Raman signals of V_N_ and V_S_ virus, and both viruses exhibited distinguishable Raman bands, as shown in Fig. 4a. V_S_ exhibited distinct Raman bands near 568, 644, 884, 950, 1027, 1310, and 1445 cm^−1^, which were also the main characterized Raman bands of SARS-CoV-2 S (Fig. 2d). This indicates that S protein on the viral surface can mainly contribute to the key characteristic Raman signals and chemical profiles of the whole virus particle [35]. In fact, SARS-CoV-2 particles are covered by S proteins with the size of several nanometers, and S protein is the key target for distinguishing different type of coronavirus. Furthermore, the simulated main EM enhancement (Fig. 4b) is localized within a 10 nm-area from the surface of nanoneedles. ACE2 with high affinity can specifically capture SARS-CoV-2 and localize only S protein of virus within the strongest-SERS area of 10 nm, leading to high-enhanced Raman signals of S protein which would overwhelm less-enhanced Raman signals of other part of virus. Therefore, this characteristic Raman signals usually contain the profile of S protein will tend to dominate the SERS spectra of coronavirus. Furthermore, we measured the SERS signals of inactivated SARS-CoV-2 virus, and the real SARS-CoV-2 virus exhibited distinct Raman bands near 645, 884, 950, 1027, 1215, and 1445 cm^−1^, which were also the main characterized Raman bands of V_S_. Consequently, it indicated that V_S_ exhibited the main characteristic Raman signals with real SARS-CoV-2, could be used to stimulate the SERS detection process for real SARS-CoV-2 virus. V_N_ exhibited distinct Raman bands (Fig. 4a) near 772, 861, 1027, 1380, and 1445 cm^−1^, which were also the main characterized Raman bands of SARS-CoV-2 nucleocapsid protein (Fig. 2d). Furthermore, these two viruses shared the same two bands at 1027 and 1445 cm^−1^, but showed different relative intensity distributions among the bands. Obviously, V_S_ and V_N_ viruses can be distinguished clearly by their SERS spectra, whereas V_S_ demonstrated distinct peak at 884 cm^−1^, and V_N_ exhibited distinct peak at 772 cm^−1^.

As we all know, the infectious SARS-CoV-2 is mostly likely to be present in the sanitary sewage discharged from some large crowd gathering places such as hospitals, communities, airports, and schools, which shed from the COVID-19 patients. In order to simulate the identification scene of contaminated water by SARS-CoV-2 virus, two kinds of viruses (V_S_ and V_N_) with different viral load were mixed in the urine of healthy eight-year-old girl, respectively. These two viruses in this healthy urine detected by ACE2-functionalized GNAs exhibited slightly different Raman bands (Fig. 4a-1,2), compared with two viruses in PBS solution detected by GNAs without ACE2-modification. The band near 884 cm^−1^ is distinct Raman characteristic of V_S_ in urine, distinguished from V_N_ in urine. Then, we built an identification standard to distinguish the clinical urine samples of healthy urine, and the urine containing V_S_ or V_N_ virus [30, 37, 38, 41, 42]. Every 100 SERS spectra obtained from multiple different samples and measuring-spots of normal urine, V_S_ in urine and V_N_ in urine as the standard SERS data to simulate normal water without virus (V_S_-negative, −), contaminated water by SARS-CoV-2 virus (V_S_-positive, +), and contaminated water with other virus. Figure 4c exhibits principal component analysis (PCA) results with distinct 95% confidence ellipses for the represented V_S_-positive urine, V_N_-positive urine, and the negative urine, as well as each ellipse does not overlap. The Raman shift wavelengths that contribute to PCs (Principal component) in positively and negatively high levels, as well as those with high and low loading values in PC loading plots, are keys to distinguish Vs-positive urine from negative (pure) urine. Subsequent loading plot analysis (SLPA, Fig. S8) highlights the key characteristic spectra peaks for V_S_-positive urine at 950, 1027, and 1445 cm^−1^ and negative urine without Vs at 677 and 900 cm^−1^, respectively. Therefore, the bands at 950 and 1027 cm^−1^ and the ratio of peak intensity of viral surface S protein can be highlighted as the discriminants between normal negative-urine and V_S_-positive urine. Furthermore, SLPA indicates the key characteristic spectra peaks for Vs at 950 and 1310 cm^−1^ and V_N_ at 772 and 1027 cm^−1^, respectively (Fig. S9). This result highlights that PCA is not only helpful to distinguish whether the coronavirus is presented in the urine sample, but also beneficial to identify which viral envelope protein is specifically expressed on the infected coronavirus.

### Validation the SARS-CoV-2 Identification Standard Based on Machine Learning Method

Next, we validated the SARS-CoV-2 identification standard based on ACE2-functionalized-GNAs as SERS probes. To simulate more complex multi-proteins-containing contaminated water detecting scene, this standard was validated by detecting the virus in one healthy adult urine and one chronic nephritis urine. Normal adult urine contains water (95%), urea (1.8%), uric acid (0.05%), mineral salts (1.1%), traces of protein, glucose, exfoliated cells, and extracellular vesicles. Besides above substances, higher content of proteins such as albumin, haemoglobin can be identified from the chronic nephritis’ urine. If SARS-CoV-2 can be selectively captured and identified from the chronic nephritis’ urine with so complex circumstance containing multi-kinds of proteins and vesicles by our ACE2-functionalized GNAs, it will have practical application in identification of the contaminated water environment by SARS-CoV-2 virus. Figure 4d exhibits DA (Discrimination Analysis) results to identify the clinical urines for chronic nephritis with the protein of 200 mg/1000 mL (24 h protein quantification), and V_S_-containing chronic nephritis. We can find that 100 SERS spectra for chronic nephritis’ urines can be attributed to negative urine (−), while 100 SERS spectra for V_S_-containing chronic nephritis’ urines can be correctly attributed to V_S_-positive (**+**), demonstrating our ACE2-functionalized GNAs can accurately capture Vs virus from complex multi-proteins urine and it is effective to distinguish whether the chronic nephritis are infected by V_S_ virus (Table 1). Furthermore, to demonstrate the selective capturing-coronavirus ability of ACE2-functionalized GNAs, the adult urine mixed both V_S_ and V_N_ virus was identified with DA method, as shown in Fig. 4e. All of 100 samples mixed with V_S_ and V_N_ virus can be attributed to V_S_-positive, demonstrating it is effective to selectively capture Vs virus with SARS-CoV-2 S on the surface from multi-virus complex circumstance.

**Table 1:** DA results to identify V_S_-containing adult urines, V_S_-containing chronic nephritis’s urines, and Very-low-titer V_S_-containing adult urines

Samples	Number of samples	Viral load (copies mL^−1^)	Identification results	Detection rate (%)	Figures
Chronic nephritis urine	100	0	100 (−)	100	Figure 4d
V_S_-containing chorionic nephritis urine	100	2200	100 (+)	100	Figure 4d
V_S_-containing adult urine	6	2200	6 (+)	100	Fig. S10
V_S_-containing chronic nephritis urine	6	2200	6 (+)	100	Fig. S10
V_S_-containing adult urine	9	220	5 (−) 4 (+)	44.4	Fig. S10
V_S_ and V_N_ mixed urine	100	2200	100(+)	100	Figure 4e
V_S_-containing adult urine	1	80	1(+)	100.0	Figures 4f and S12

### Detection Limit of ACE2-functionalized-GNAs Down to Single-virus Level

In order to evaluate the LOD of SERS-chips for detecting virus, DA results to identify V_S_-containing adult urines, V_S_-containing chronic nephritis’s urines and low-titer V_S_-containing adult urines, were shown in Fig. S10. All 6 SERS spectra for V_S_-containing adult urines, and all 6 SERS spectra for V_S_-containing chronic nephritis’s urines can be attributed to V_S_-positive (**+**), demonstrating it is effective to distinguish V_S_ from the complex multi-proteins-containing contaminated water environment. Until the viral load of urine samples was lowered to 220 copies mL^−1^, four samples were still correctly attributed to V_S_-positive (**+**) in all nine samples. In fact, the maximum of virus particles averagely distributed in a Raman-focused window was evaluated to be 0.6 (details, see S4), indicating the single-virus-detection ability where the measuring area could be correctly identified as long as there was only single coronavirus in this area. Even the viral load in urine was down to 80 copies mL^−1^, while we applied SERS mapping (40 × 30 μm^2^) and checked 300 measuring area for one urine sample (Fig. S12), 42 dot-measuring-areas were identified as V_S_-positive (Fig. 4f). It demonstrated that 42 viral particles had been identified (**+**), and this urine sample could be identified as positive, although the checking time was more nearly 5 min from the normal measuring time of several seconds. Meanwhile, it also indicated that around 70% of Vs in urine samples could be captured by the synergistic effect of the high affinity of ACE2 with S protein and the as-designed “virus-traps” nanoforest (details, see S5). Therefore, the LOD of our ACE2-functionalized SERS chips for detection of Vs virus is as low as 80 copies mL^−1^, which can parallel the sensitivity of RT-PCR detection method. In summary, the SERS mapping detection method with the scanning area (≥ 40 × 30 μm^2^, 5 min) is recommended to detect coronavirus-containing clinical samples in order to decrease the false-negative rate, although single-point SERS detection within several seconds can be used to detect the clinical samples with the SARS-CoV-2 viral load larger than 2200 copies mL^−1^. In addition, in the face of complex, remote, and diverse outdoor detecting scenes, SERS detection of virus shows more convenient, rapidly, and economical characteristics, which can satisfy the needs for monitoring and early warning of SARS-CoV-2 in outdoor contaminated water environments.

## Conclusions

In conclusion, we have developed an ACE2-functionalized gold “virus-traps” nanoarray as a novel COVID-19 SERS sensor to capture and identify SARS-CoV-2-like virus with extremely high sensitivity and specificity. ACE2 with high affinity to S protein can specifically capture SARS-CoV-2 virus and localize only viral surface S protein within a 10 nm strong electromagnetic-field enhanced area from the nanoneedles surface, where the strongest-SERS enhancement including “lighting-rod” and “hot-spots” effects can boost unique highly enhanced Raman signals of S protein to represent SARS-CoV-2 virus. Such a SERS sensor features extraordinary 10^6^-fold virus enrichment and 10^9^-fold enhancement of SERS signal, resulting in the detection limit down to single-virus level. The identification standard of SERS signals established by machine-learning and identification techniques has been utilized to identify simulated COVID-19 from urines with the viral load of as low as 80 copies mL^−1^ as short as 5 min, which is of great significance for achieving real-time monitoring and early warning of coronavirus. More importantly, the developed ACE2-functionalized SERS sensor and machine-learning and identification standard is an extendable detection method, which is capable of accurately capturing yet-unknown coronaviruses as long as it’s S-protein can combine with ACE2 protein. The strategy developed here can be used to quickly establish the identification standard based on their SERS spectra of the emerging coronaviruses and machine-learning techniques, and immediately enable extremely sensitive and rapid detection of novel virus, which provides the first paradigm of breakthrough of applying SERS technology in the virus detection field.

## Supplementary Information

Below is the link to the electronic supplementary material.

Supplementary file1 (MP4 442 KB)

Supplementary file2 (PDF 1407kb)
