# Supplementary material for: Human ACE2-Functionalized Gold “Virus-Trap” Nanostructures for Accurate Capture of SARS-CoV-2 and Single-Virus SERS Detection
Source: Nanomicro Lett. 2021 Apr 13;13:109. doi: 10.1007/s40820-021-00620-8 (PMC8042470; doi:10.1007/s40820-021-00620-8)
Supplement: Supplementary file 2 — Supplementary file2 (PDF 1407kb) [file 40820_2021_620_MOESM2_ESM.pdf]

Supporting Information for

Human ACE2-Functionalized Gold "Virus-Trap" Nanostructures for Accurate Capture of SARS-CoV-2 and Single-Virus SERS Detection

Yong Yang [1, 2, 3, *, #], Yusi Peng [1, 2, #], Chenglong Lin [1, 2], Li Long [4], Jingying Hu [5], Jun He [6, 7, *], Hui Zeng [8], Zhengren Huang [1, *], Zhi-Yuan Li [4], Masaki Tanemura [9], Jianlin Shi [1], John R. Lombardi [10, *], Xiaoying Luo [5, *]

[1]State Key Laboratory of High-Performance Ceramics and Superfine Microstructures, Shanghai Institute of Ceramics, Chinese Academy of Sciences, 1295 Dingxi Road, Shanghai 200050, P. R. China

[2]Graduate School of the Chinese Academy of Sciences, Beijing 100049, P. R. China

[3]Center of Materials Science and Optoelectronics Engineering, University of Chinese Academy of Sciences, Beijing 100049, P. R. China

[4]School of Physics and Optoelectronics, South China University of Technology, Guangzhou 510640, P. R. China

[5]State Key Laboratory of Oncogenes and Related Genes, Shanghai Cancer Institute, Renji Hospital, Shanghai Jiaotong University School of Medicine, 200032, Shanghai, P. R. China

[6]Anhui Provincial Center for Disease Control and Prevention, 12560, Hefei, Anhui, P. R. China

[7]Public Health Research Institute of Anhui Province, 12560, Hefei, Anhui, P. R. China

[8]Shanghai Yangpu Hospital of Traditional Chinese Medicine, Shanghai 200090, **P. R.** China

[9]Department of Frontier Materials, Nagoya Institute of Technology, 466-8555, Nagoya, Japan

[10]Department of Chemistry, The City College of New York, 160 Convent Avenue, New York, NY 10031, USA

[#] Yong Yang and Yusi Peng contributed equally to this work

*Corresponding authors. E-mail: yangyong@mail.sic.ac.cn (Yong Yang), heliosking@sina.com (Jun He), zhrhuang@mail.sic.ac.cn (Zhengren Huang), jlombardi@ccny.cuny.edu (John R. Lombardi), luoxy@shsci.org (Xiaoying Luo)

S1 SERS Enhancement Factors of GNAs

The samples for SERS measurement were prepared by soaking the sample into R6G ethanol solutions with different concentrations for 2 h, then rinsing in ethanol and drying in a gentle N_2 flow. And Raman spectra of 1 mM and 0.1 M R6G adsorbed on silicon substrates, 0.1 mM R6G

adsorbed on Au film, 1 nM and 0.1 nM R6G adsorbed on GNAs were shown in Fig. S1. To estimate the enhancement factor for our nanoneedles arrays, the total SERS substrate EF (Enhancement Factor) was calculated for R6G on the Au nanoneedles samples according to the equation as follows:

$$TSSEF = \frac{I_{surf}/N_{surf}}{I_{bulk}/N_{bulk}} \quad \text{(S1)}$$

where I_{surf} and I_{bulk} denote the integrated intensities for 1365 cm^{-1} band of the 0.1 nM R6G adsorbed on the GNAs and 0.1 M R6G on silicon respectively. Whereas N_{surf} represent the corresponding total number of R6G molecules excited by the laser beam, which include molecules adsorbed on Au nanoneedles and adsorbed elsewhere on the surface in between Au nanoneedles. N_{bulk} represent the average number of R6G molecules on silicon in the scattering volume for the Raman measurement. As for the numerator ratio of molecules in a in a Raman-focused window on the GNAs and silicon substrates is shown in follows:

$$\frac{N_{GNAs}}{N_{Si}} = \frac{S_{GNAs}}{S_{Si}} = \frac{3.14 \times 1^2 + 9 \times 3.14 \times 0.15^2 + 3.14 \times 0.3 \times 0.5 \times 0.7 \times 9 \times 3.14}{3.14 \times 1^2} = 3.8$$

In this formula, the diameter of the nanoneedles in the stem part is around 300 nm, and the density of the nanoneedles is approximately 9 μm^{-2}. The axis lengths are around 700 nm, and the lengths of the nanoneedles is 300 nm. The diameter of the focused area for Raman measurements is 2 μm. Therefore, the number of molecules in a in a Raman-focused window on the GNAs substrates is 3.8 times that of silicon substrates for the same concentration of probe molecule solution, the value of N_{surf}/N_{bulk} is determined to 3.8×10^{-8}. Therefore, it is noted that an enhancement factor of above 3.9×10^9 for GNAs was obtained relative to silicon substrates.

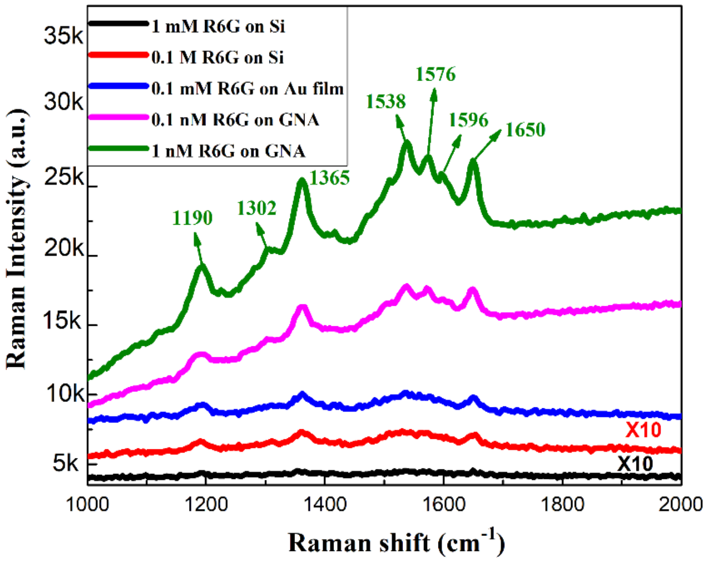

Fig. S1 Raman spectra of 1 mM and 0.1 M R6G adsorbed on silicon substrates, 0.1 mM R6G adsorbed on Au film, 1 nM and 0.1 nM R6G adsorbed on GNAs substrates

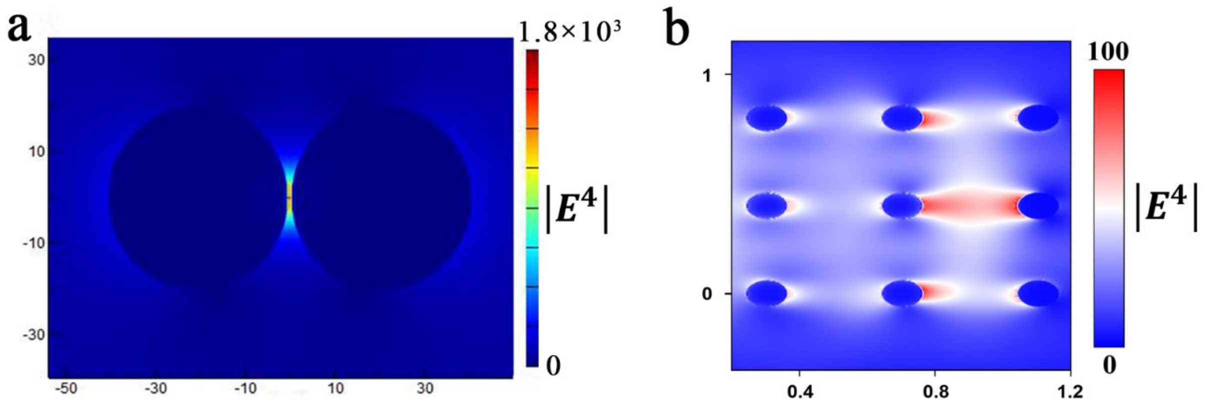

Fig. S2 Calculated intensity distribution (|E^4|) at 785 nm for **a** two adjacent nanoparticles with 40 nm diameter and **b** a tilted Au nanoneedle array with the polarization of the incident laser along the x-axis

S2 Introduction for SARS-CoV-2 Virus and Related Raman Character

SARS-CoV-2 and SARS-CoV are belong to betacoronavirus, have single-positive strand RNA genome [1, 2]. CoV genomes encode four structural proteins: spike (S), envelope (E), matrix (M), and nucleocapsid (N), and Spike proteins are overlapped on the surface of CoV. Recent report demonstrated that SARS-CoV-2 and SARS-CoV S share the same functional host-cell receptor-angiotensin-converting enzyme 2 (ACE2) [3]. The higher affinity of SARS-Cov-2 S (10- to 20-fold than that of SARS-CoV) for Human ACE2 may contribute to the significant ease with which SARS-CoV-2 can spread from person-to-person [S1].

Surface protein and lipid profiles are distinctive characteristics of each virus. Usually, viruses can generate detectable characteristic Raman signals containing the surface protein and lipid profiles when their individual surface molecules are adequately in contact with metal nanoparticles, because viruses possess unique surface protein and lipid profiles on their outer layer [4, 5]. SARS-CoV-2 viruses are approximately 100 nm in diameter and thereby larger than the molecules that are conventionally analysable by SERS. In the case of SARS-CoV-2, the spike glycoprotein (S protein) on the virion surface mediates receptor recognition and membrane fusion [S1, S3]. CoV-2 virus particles are covered by S proteins with the size of several nanometer. When their individual surface of SARS-CoV-2 virus are adequately in contact with metal nanoparticles, the surface S proteins will range in the “hot-spots” area and generate detectable characteristic Raman signals. Therefore, the detectable characteristic Raman signals usually contained the surface S protein will tend to dominate the SERS-Raman spectra of coronavirus. Consequently, we believed that real-time coronavirus detection can be achieved by measuring the Raman signals from the surface molecules of coronavirus.

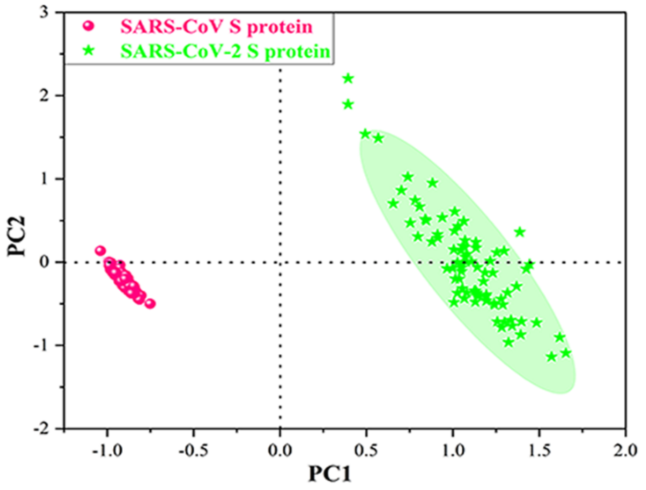

Fig. S3 PCA analysis results of SARS-CoV-2 S protein and SARS-CoV S protein

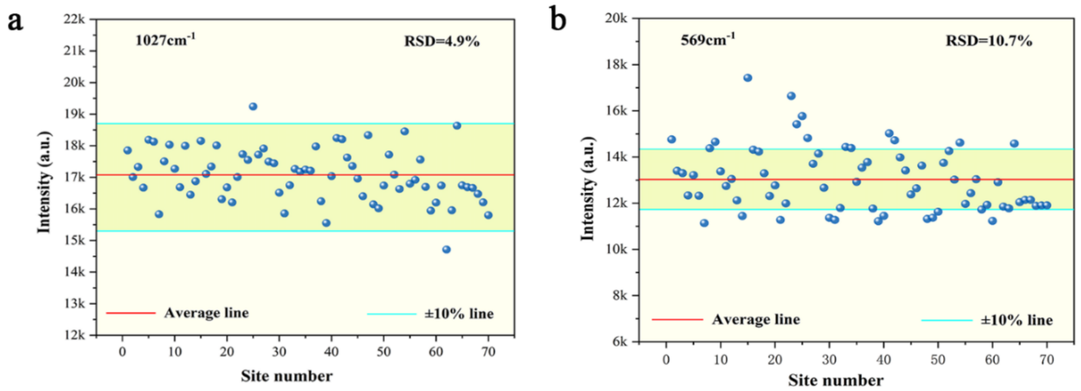

Fig. S4 Raman signals of SARS-CoV-2 S protein collected from 70 selected points on the GNAs substrates at 1027 cm^{-1} **a** and 569 cm^{-1} **b** with the area of 20×14 μm^2

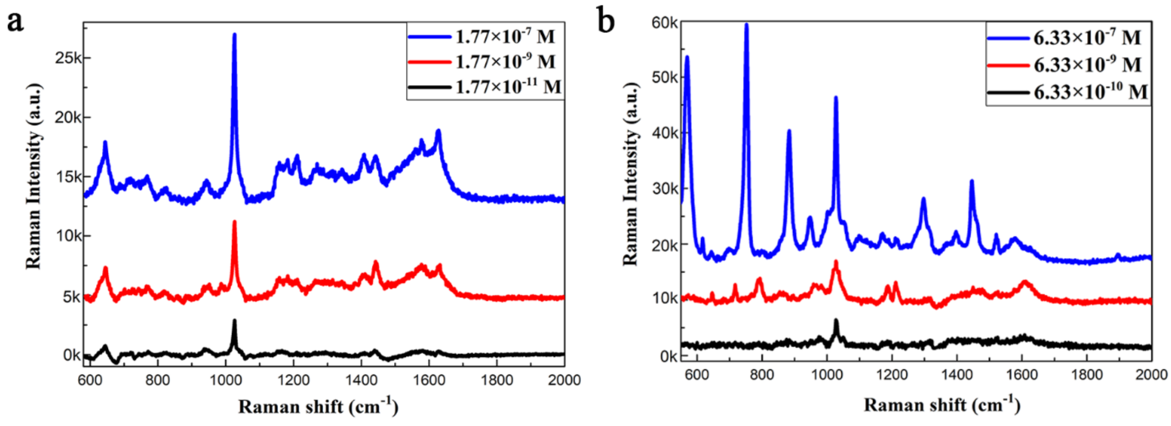

Fig. S5 a Limit of detecting SARS-CoV-2 S with ACE2-modified GNAs by immersing in the diluted protein solution. **b** Limit of detecting SARS-CoV-2 S without ACE2-modified GNAs by dropping diluted protein solution. It is worth noting that the application of dropping method is because GNAs without ACE2 modification have little affinity with SARS-CoV-2 S protein, and can't obtain the Raman signals for such-low concentration R6G by immersing method.

Table S1 Raman peak assignments for proteins and virus on GNAs substrates

**Assignments (Literature)**	**Reference Literature /(cm^{-1})**	**Experiment Au /(cm^{-1})**	**Calculation Au / (cm^{-1})**	**Assignments (Calculation results)**
Protonated amine group, δ(-NH_3^+)	Ref [8]	454	439-452	Amine group, δ(-NH_2)
Amide V	567 [7]	568	547-583	bre (ϕ) & v(C-C, Skeleton)
Tyr, $\rho_{\tau,}$(C-C)	645 [6]	646	649	Tyr, $\rho_{\tau,}$(C-C)
Trp, v(ϕ)	758 [6]	752	742-771	Phe & Trp, v(ϕ, indole ring)
Trp, W17 [δ$(CH)_{\phi Ph}$ & δ(N-H)]	880 [7] 874 [8]	884	865-889	δ(N-H)
Skeleton, v(C-C, α-Helix)	Ref [8]	950	935-954	Skeleton, v(C-C)
Phe, $\rho_{ipb,}$(C-H)	1034 [8] 1022 [7]	1027	1017-1038	δ & v (C=C, -CH) in aromatic ring
Trp & Phe, v(C-C_6H_5)	1209 [6]	1214	1232	Phe & Tyr, v(C-C_6H_5)
Amide III (α-Helix)	Ref [9]	1296	1266-1270	Skeleton, v(C-H) & v(N-H)
Trp, W7 [v(N_1–C_8)]	1365 [7]	1380	1377-1388	Skeleton, v(-CH2) & v(-C-C=O) & v(C-H)
Amino acid, v(-CH_2)	Ref [6, 8]	1445	1415-1443	Amino acid, v(-CH_2)
Amide II	1515 [9] 1509-1552, 1529 [10]	1521	1501-1531	$\rho_{\tau,}$(C-H) & v (C=N) & v(C=C)
Tyr & Phe, v(ϕ)	1591 [8]	1592	1591-1599	v(ϕ) or v(indole ring)
v(C=C)	1617-1680 [6]	1626	1624-1656	Phe, Trp, Tyr, v(ϕ) & δ(ϕ)
CH、CH_2&CH_3 of aliphatic group, v(C-H)	2890-2980 [6]	2890, 2950	3040-3210	Skeleton, v(C-H) & v(N-H)

Abbreviation: δ, deformation; v, stretching; $\rho_{\tau,}$ *twisting vibrations;* ϕ*, aromatic ring;* $\rho_{ipb,}$ *in-plane deformation;*

S3 Calculation of Enrichment Multiples

As for the SARS-CoV-2 S protein of Fc-Tag (2.38 mg/mL, 20 μg/tube, M= 75,243), the number of mole (n) and molecules (N) are shown below:

$n=m/M=20\times10^{-6}\ g/75243=2.66\times10^{-10}$ mol

$N=n\times N_A=2.66\times10^{-10}\times 6.02\times10^{23}=1.6\times10^{14}$

When the SARS-CoV-2 S protein solution was diluted to 1.5 mL, the concentration of solution is shown below:

C_1=n/V=2.66 $\times 10^{-10}/(1.5 \times 10^{-3})$=1.77 $\times 10^{-7}$ M

Then, the SARS-CoV-2 S protein solution was further diluted to reach at the LOD of 1.77×10^{-11} M. When the Raman detecting of SARS-CoV-2 S with ACE2-modified GNAs (5 mm×5 mm) was conducted by immersing in the diluted protein solution of 1.77×10^{-11} M (LOD), the number of virus (n_1) in a Raman-focused window without enrichment of virus is shown below:

$$n_1 = 5\,mm \times 5\,mm \times 5\,nm \times 1.77 \times 10^{-11} \times 6.02 \times 10^{23} \times 3.14 \times \frac{1\,\mu m^2}{5\,mm^2} \approx 1.7 \times 10^{-4}$$

In this formula, the size of proteins usually ranged from 1 nm to 10 nm, and the value of proteins' diameter is determined to 5 nm here. The diameter of the focused area for Raman measurements is 2 μm. The presence of the S protein Raman signal indicates the at least one protein is exsited in the focused area for Raman measurement.

When the SARS-CoV-2 S protein solution was further diluted to 6.33×10^{-10} M, The Raman detecting of SARS-CoV-2 S was conducted by dropping the diluted protein solution of 6.33×10^{-10} M (LOD) on ACE2-modified GNAs (5 mm×5 mm), the number of virus (n_2) in a Raman-focused window without enrichment of virus is shown below:

$$n_2 = 10 \times 10^{-6} \times 6.33 \times 10^{-10} \times 6.02 \times 10^{23} \times 3.14 \times \frac{1\,\mu m^2}{5\times 5\,mm^2} \approx 500$$

Therefore, the enrichment multiple is $\geq \frac{500}{1.7\times 10^{-4}}$ =2.94×10^{6}, assuming the same number of molecules for LOD can be detected by immersing ACE2-modified GNAs and dropping on GNAs without ACE2-modification. It indicates that the S proteins can be enriched (10^6-fold) on GNAs by ACE2-modification.

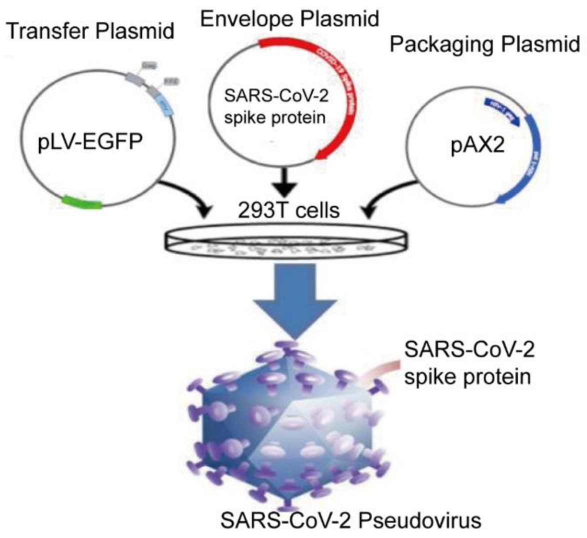

Fig. S6 Schematic diagram of the spike protein-containing SARS-CoV-2 pseudovirus

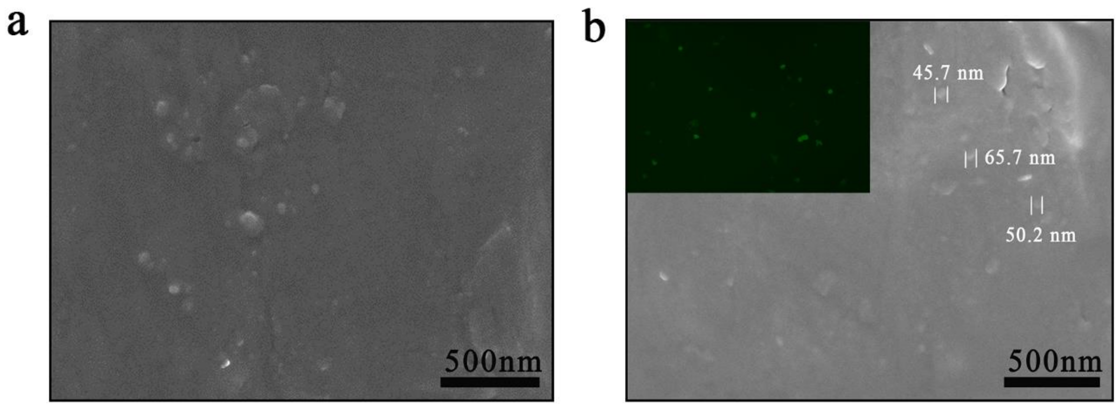

Fig. S7 Morphologies of the packaged virus particles of **a** V_N and **b** V_S were measured by scanning electron microscopy. The fluorescence image that cells was transfected by GFP-expressed V_S virus to validation the titer is shown in illustration, then V_S virus was confirmed

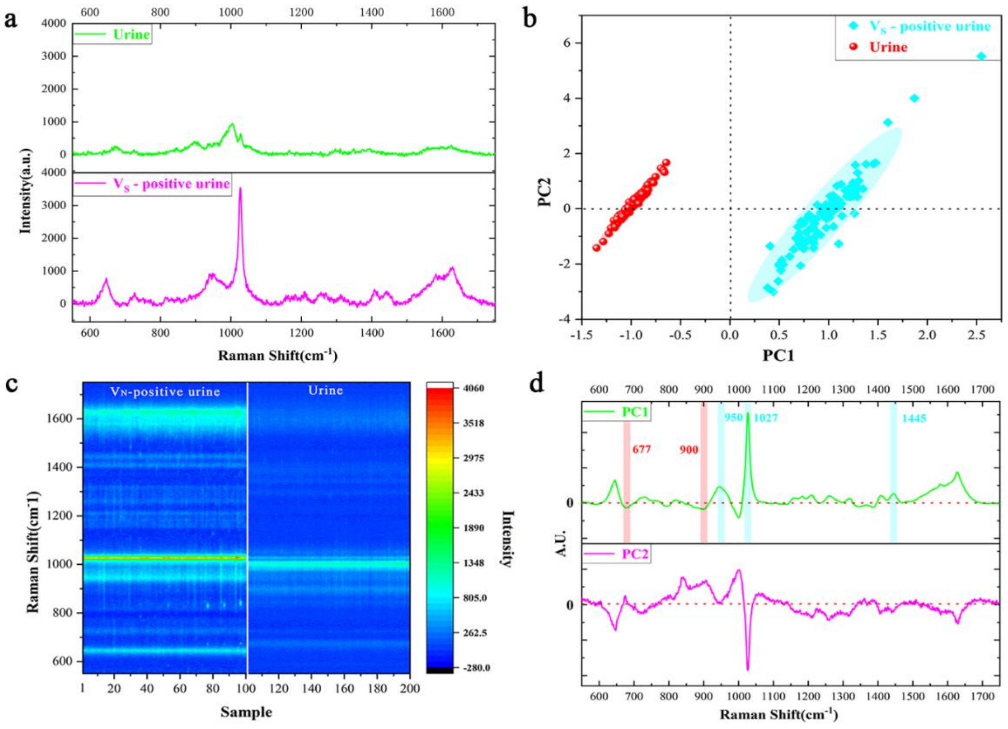

Fig. S8. a SERS spectra of healthy girl's urine and V_S in urine (2200 copies/mL). **b** 2D PCA plots for the Raman spectra from pure urine and V_S in urine. Red dots and blue squares represent the projected Raman spectra for the pure urine and V_S-positive urine. **c** The Raman intensity mapping from pure urine (right) and V_S-positive urine (left) at different frequency positions. **d** Loading of the PC1 and PC2 for the spectra from pure urine and V_S-positive urine. The green line represents the loading of PC1, and the purple line represents the loading of PC2. The PCA result shows that the SERS for urines are uniquely projected to the left for negative urine (PC1 < 0, Fig. S7-b) and right for V_S–positive urine (PC1 > 0, Fig. S7-b). The pink and blue bands indicate the characteristic Raman shifts for pure urine and V_S-positive urine, respectively.

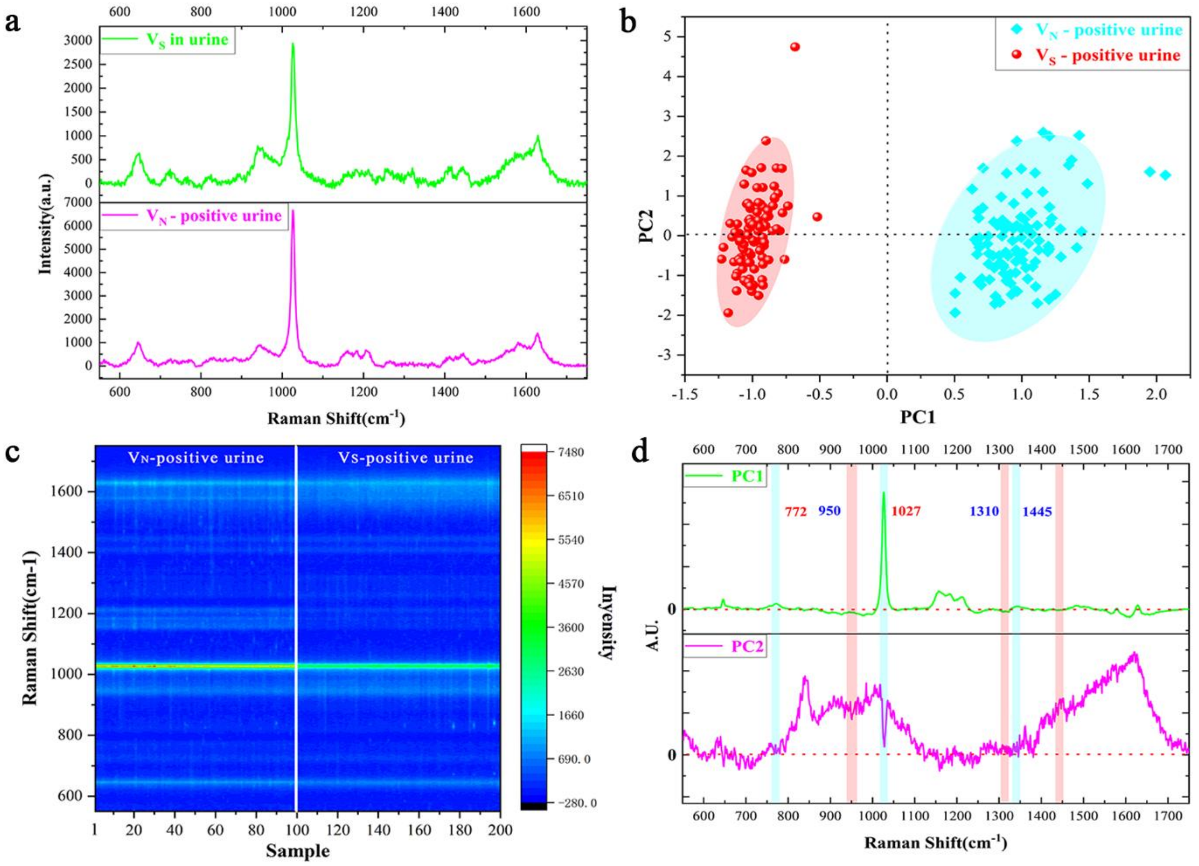

Fig. S9 a SERS spectra of V_S-positive and V_N-positive urines (2200 copies/mL). **b** 2D PCA plots for the Raman spectra from V_S-positive and V_N-positive urines. Red dots and blue squares represent the projected Raman spectra for V_S-positive and V_N-positive urines. **c** The Raman intensities mapping from V_N-positive (left) and V_S-positive urine (right) at different frequency positions. **d** Loading of the PC1 and PC2 for the spectra from V_S-positive and V_N-positive urines, the green line represents the loading of PC1, and the purple line represents the loading of PC2. The PCA result shows that the SERS for urines are uniquely projected to the left for V_S–positive urine (PC1 < 0, Fig. S8-b) and right for V_N–positive urine (PC1 > 0, Fig. S8-b). The pink and blue bands indicate the characteristic Raman shifts for V_S-positive and V_N-positive urines, respectively.

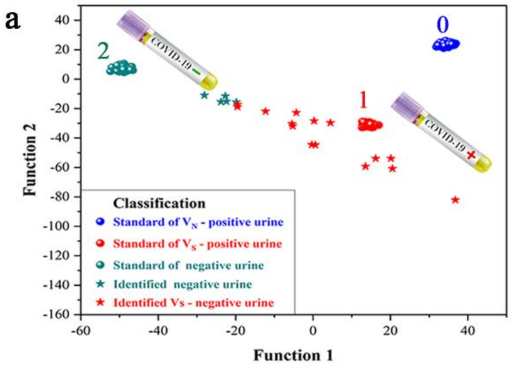

b

Classification Results[a]

			Predicted Group Membership 0	1	2	Total
Original	Count	0	100	0	0	100
		1	0	100	0	100
		2	0	0	100	100
		Ungrouped cases	0	16	5	21
	%	0	100.0	.0	.0	100.0
		1	.0	100.0	.0	100.0
		2	.0	.0	100.0	100.0
		Ungrouped cases	.0	76.2	23.8	100.0

a. 100.0% of original grouped cases correctly classified.

Fig. S10 DA results to identify 6 Vs-containing adult urines, 6 Vs-containing chronic nephritis' urines with the viral load of 2200 copies/mL, and 9 Vs-containing adult urines with the viral load of 220 copies/mL

S4 Maximum of Virus Particles in a Raman-focused Window for Measuring V_S-containing Urine with the Viral Load of 220 Copies/mL

The SERS chips (0.5×0.5 mm^2) was conducted to collect the Vs virus by operation as Fig. S8. V_S-containing urine of 200 mL (stimulated the volume of normal adult urine each time, about 100-300 mL) with the viral load of 220 copies/mL were operated 2-3 cycles to enable the virus bind with SERS-chips as completely as possible. The diameter of the focused area for Raman measurements is 2 μm. Therefore, the maximum of average distributed virus particles in a Raman-focused window is as follow:

$$220\, copies/mL \times 200\, mL \times \frac{3.14 \times 1\mu m^2}{500\ \mu m \times 500\ \mu m} \approx 0.6$$

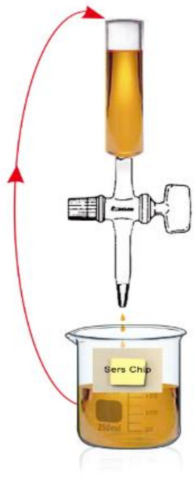

Fig. S11 Scheme for the SERS chips for collecting very-low-viral-load urines

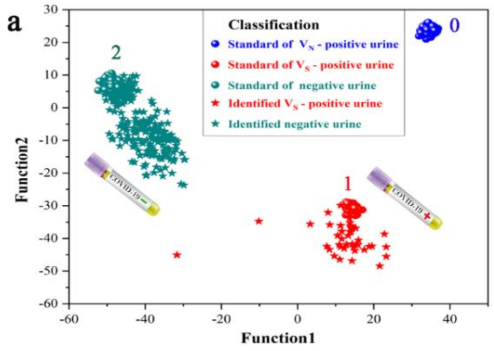

b

Classification Results[a]

			Predicted Group Membership			
			0	1	2	Total
Original	Count	0	100	0	0	100
		1	0	100	0	100
		2	0	0	100	100
		Ungrouped cases	0	42	258	300
	%	0	100.0	.0	.0	100.0
		1	.0	100.0	.0	100.0
		2	.0	.0	100.0	100.0
		Ungrouped cases	.0	14.0	86.0	100.0

a. 100.0% of original grouped cases correctly classified.

Fig. S12 DA results to identify V_S-containing adult urine with the viral load of 80 copies/mL. The 300 measuring points for one urine sample were checked by SERS mapping (40 μm×30 μm). The green, red, blue balls represent the standard of V_S-negative urine, the standard of V_S-positive urine, the standard of V_N-positive urine. The green and red star represent identified V_S-negative urine and identified V_S-positive urine.

S5 Capture Efficiency of Vs

If all virus in V_S-containing urine of 200 mL with the viral load of 80 copies/mL were absorbed on the SERS chips with area of 0.5 mm×0.5 mm, the maximum of average distributed virus particles in a Raman-focused window is as follow:

$$80\, copies/mL \times 200\, mL \times \frac{3.14 \times 1\mu m^2}{500\ \mu m \times 500\ \mu m} \approx 0.2$$

According to the DA results of V_S-containing adult urine with the viral load of 80 copies/mL, the 300 measuring points for one urine sample were checked by SERS mapping and 42 V_S-positive urine points were identified, which indicated that the actual detected rate of V_S in a Raman-focused window is 0.14. Therefore, the capture efficiency (η_1) of Vs in urine samples on GANs is as follow:

$$\eta_1 = \frac{0.14}{0.2} = 70\%$$

Supplementary References

[S1] D. Wrapp, N. S. Wang, K. S. Corbett, J. A. Goldsmith, C. L. Hsieh et al., Cryo-EM Structure of the 2019-nCoV spike in the Prefusion Conformation. Science **367,** 1260-1263 (2020). http://science.sciencemag.org/content/367/6483/1260

[S2] M. Hoffmann, K. W. Hannah, S. Schroeder, N. Kruger, T. Herrler et al., SARS-CoV-2 Cell Entry Depends on ACE2 and TMPRSS2 and Is Blocked by a Clinically Proven Protease Inhibitor. Cell **181**(2), 271-280 (2020). https://doi.org/10.1016/j.cell.2020.02.052

[S3] R. Tuma1, G. J. Thomas, *Raman Spectroscopy of Viruses* (Handbook of Vibrational Spectroscopy, 2006).

[S4] D. Němĕcek and G. J. Thomas, *Raman Spectroscopy in Virus Structure Analysis* (Handbook of Molecular Biophysics. Methods and Applications, 2009).

[S5] M. Keshavarz, B. Tang and K. Venkatakrishnan, Label-Free SERS Quantum Semiconductor Probe for Molecular-Level and in Vitro Cellular Detection: A Noble-Metal-Free Methodology. ACS Appl. Mater. Interface **10,** 34886−34904 (2018). https://doi.org/10.1021/acsami.8b10590

[S6] K. L. Aubrey, G. J. Thomas, Raman spectroscopy of filamentous bacteriophage Ff (fd, M13, f1) incorporating specifically-deuterated alanine and tryptophan side chains. Assignments and structural interpretation. Biophys. J. **60**(6), 1337-1349 (1992). https://doi.org/10.1016/S0006-3495(91)82171-3

[S7] S. Krimm and J. Bandekar, Vibrational Spectroscopy and Conformation of Peptides, Polypeptides, and Proteins. Adv. Protein Chem. **38**(C), 181-364 (1986). https://doi.org/10.1016/S0065-3233(08)60528-8

[S8] T. Miura, G. J. Thomas, Raman Spectroscopy of Proteins and Their Assemblies. Subcell. Biochem. **24,** 55-99 (1995). https://doi.org/10.1007/978-1-4899-1727-0_3

[S9] J. Bandekar, Amide Modes and Protein Conformation. Biochim. Biophys. Acta **1120**(2), 123-143 (1992). https://doi.org/10.1016/0167-4838(92)90261-B

[S10] M. Cardona, *Light Scattering in Solids II*, Chapter 2 Resonance Phenomena. (Springer Berlin Heidelberg, 1982).
